# Giant magnetoresistance ratio in a current-perpendicular-to-plane spin valve based on an inverse Heusler alloy Ti_2_NiAl

**DOI:** 10.3762/bjnano.10.161

**Published:** 2019-08-08

**Authors:** Yu Feng, Zhou Cui, Bo Wu, Jianwei Li, Hongkuan Yuan, Hong Chen

**Affiliations:** 1 School of Physics and Electronic Engineering, Jiangsu Normal University, Xuzhou 221116, People’s Republic of China; 2 School of Physical Science and Technology, Southwest University, Chongqing 400715, People’s Republic of China; 3 Department of Physics, Zunyi Normal College, Zunyi 563002, People’s Republic of China

**Keywords:** current-perpendicular-to-plane geometry, Heusler alloy, nonequilibrium Green’s function, spin transport, spintronics, spin valve

## Abstract

A Ti_2_NiAl inverse Heusler alloy based current-perpendicular-to-plane (CPP) spin valve (SV) with various kinds of atomic terminated interfaces has been designed to explore the potential application of Heusler alloys in spintronics devices. By performing first principles calculations combined with the nonequilibrium Green’s function, it is revealed that spin magnetic moments of interfacial atoms suffer a decrease, and the electronic structure shows that the TiNi^B^-terminated structure possesses the largest interface spin polarization of ≈55%. Our study on spin-transport properties indicates that the total transmission coefficient at the Fermi level mainly comes from the contribution from the spin up electrons, which are regarded as the majority of the spin electrons. When the two electrodes of the CPP-SV device are in parallel magnetization configuration, the interface containing Ti and Ni atoms possesses a higher spin up transmission coefficient than the interface containing Ti and Al atoms. The device with the TiNi^B^-terminated interface possesses the largest magnetoresistance ratio of 3.28 × 10^5^, and it has great application potential in spintronics devices.

## Introduction

Since the first theoretical prediction of the half metallicity of Heusler alloys [1], there has been explosive interest in manipulating the electron spin in a Heusler-alloy-based spintronic device [2–4]. As one of the most important spintronics devices, a current-perpendicular-to-plane (CPP) spin valve (SV) based on the giant magnetoresistive (GMR) effect consists of a nonmagnetic metal as a spacer that is sandwiched between two ferromagnetic materials. It can produce two distinct states: a low-resistance state, when two electrodes are in parallel magnetization configuration, and a high-resistance state, when they are in antiparallel magnetization configuration. Half-metallic Heusler alloys (HMHAs) are regarded as one of the most promising candidates for electrode materials in CPP-SV owing to their high Curie temperature, tunable electronic structure and small lattice mismatch with Ag or Cu. Moreover, the majority spin bands of HMHA across the Fermi level show typical metallicity, while minority spin bands possess an energy gap around the Fermi level. Such a novel band structure results in a theoretical 100% spin polarization, which is one of the most crucial parameters for CPP-SV according to the Valet–Fert model [5]. As one of the subfamilies of Heusler alloys, conventional Heusler alloys with space group FM-3M have a chemical formula of X_2_YZ where the X atom locates at (0, 0, 0) and (0.5, 0.5, 0.5) sites, and the Y and Z atoms sit at (0.25, 0.25, 0.25) and (0.75, 0.75, 0.75) sites. From an experimental point of view, a superconducting spin-valve effect has been demonstrated in a Co_2_Cr_1−_*_x_*Fe*_x_*Al-based spin valve [6]. A CPP-SV using Co_2_Mn(Ga_0.25_Ge_0.75_) has been verified to have a high resistance–area product (Δ*RA*) of 6.1 mΩ·μm^2^ and magnetoresistance (MR) ratio of 40.2% [7]. A Co_2_Fe(Ge_0.5_Ga_0.5_) [8] based CPP-SV obtained a higher Δ*RA* of 26.4 mΩ·μm^2^ and a MR ratio of 129.1% [9]. Several CPP-SVs have employed conventional Heusler alloys such as Co_2_Fe_0.4_Mn_0.6_Si [10] and Co_2_MnSi [11], also reaching a high MR ratio. On the other hand, a state-of-the-art theoretical approach that combines first principles calculations with the Keldysh nonequilibrium Green’s function theory is also an effective way to study the transport properties of a device. A high MR ratio of 174% was reported in a recent work on Fe_4_N-based CPP-SV, and its spin-polarized quantum transport properties were investigated [12]. The CoFeMnSi-based heterostructure exhibited an ultrahigh tunnel magnetoresistance (TMR) ratio of 2 × 10^3^ [13]. Nonequilibrium spin injection in a MnAl-based spintronics device was studied, and a TMR ratio of 2000% was predicted under a high bias voltage [14]. A large TMR ratio and spin Seebeck effect were found in a Ti_2_MnAl-based heterostructure [15]. Although some relatively high MR ratios have been predicted in CPP-SV devices, there is more room for further improvement in the MR value by using better half-metallic Heusler compounds. In addition to conventional Heusler compounds, much attention has been paid to a new subfamily of Heusler compounds, i.e., inverse Heusler compounds which have a space group of F-43M and chemical formula of X_2_YZ where the X atom locates at (0, 0, 0) and (0.25, 0.25, 0.25) sites, and the Y and Z atoms sit at (0.5, 0.5, 0.5) and (0.75, 0.75, 0.75 sites [16–18]. Half-metallicity has been predicted in Sc_2_MnSi [19], Ti_2_RuSn [20], and Ti_2_NiAl [21]. Spin gapless semiconductor characteristics are also demonstrated in Mn_2_CoAl [22–25] and Ti_2_MnAl [26–27]. The interface characteristics of heterostructures based on inverse Heusler alloys have been studied in detail [28–30]. Therefore, inverse Heusler compounds exhibit exceptional electronic structure and magnetic properties, and they deserve to be further studied and applied in spintronics devices.

In this study, we built a CPP-SV device employing a half-metallic inverse Heusler alloy Ti_2_NiAl as the electrode and Ag as the spacer. Different atomic-terminated interfaces are considered. We performed the first-principles density functional theory combined with nonequilibrium Green’s function to investigate the interfacial electronic structure, magnetic properties and MR ratio of the device.

## Results and Discussion

**Figure 1:**
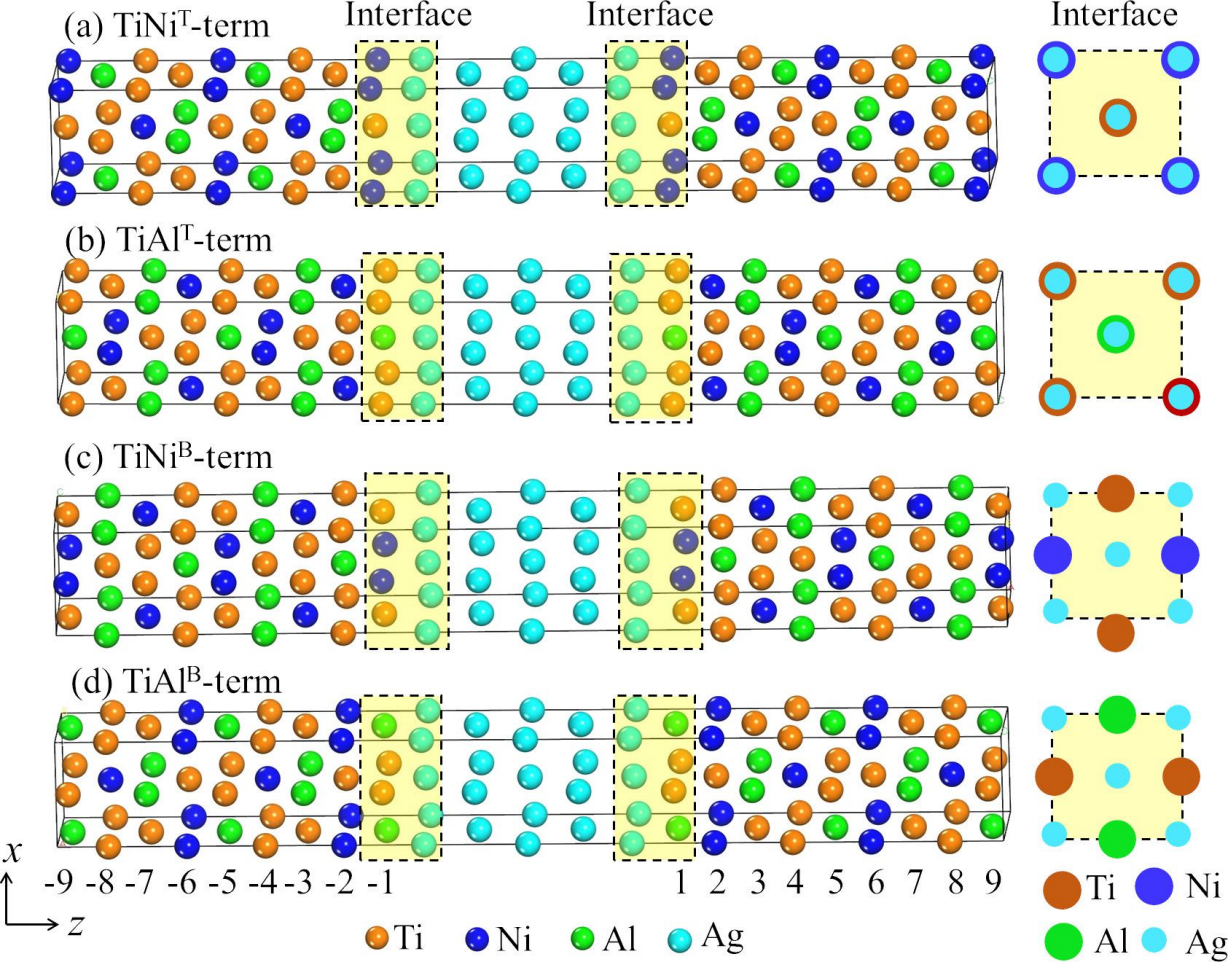
Schematic illustration of a Ti_2_NiAl/Ag/Ti_2_NiAl device with different atomic terminated interfaces. (a) TiNi^T^-terminated interface, (b)TiAl^T^-terminated interface, (c) TiNi^B^-terminated interface and (d) TiAl^B^-terminated interface.

Our investigated device is a two-probe device, where Ti_2_NiAl is employed as a semi-infinite left and right electrode, and Ag is selected to be the middle spacer layer. For bulk Ti_2_NiAl, one Ti atom locates at the (0, 0, 0) site, which is described as Ti^A^, and the other locates at the (0.25, 0.25, 0.25) site, which is described as Ti^B^, where Ni and Al sit at (0.5, 0.5, 0.5) and (0.75, 0.75, 0.75) sites. When Ti_2_NiAl is applied to the device, there are two ideal terminations along the (1 0 0) direction: TiNi and TiAl. The TiNi-terminated interface can be further categorized into two patterns: (i) a TiNi^T^-terminated interface where interfacial Ti and Ni atoms sit on the top of Ag atoms (see Figure 1a), and (ii) a TiNi^B^-terminated interface where interfacial Ti and Ni atoms locate in the bridge sites between Ag atoms (see Figure 1c). In a similar manner, the TiAl-terminated interface can be further categorized into TiAl^T^ (see Figure 1b) and TiAl^B^ (see Figure 1d) terminated interfaces.

**Figure 2:**
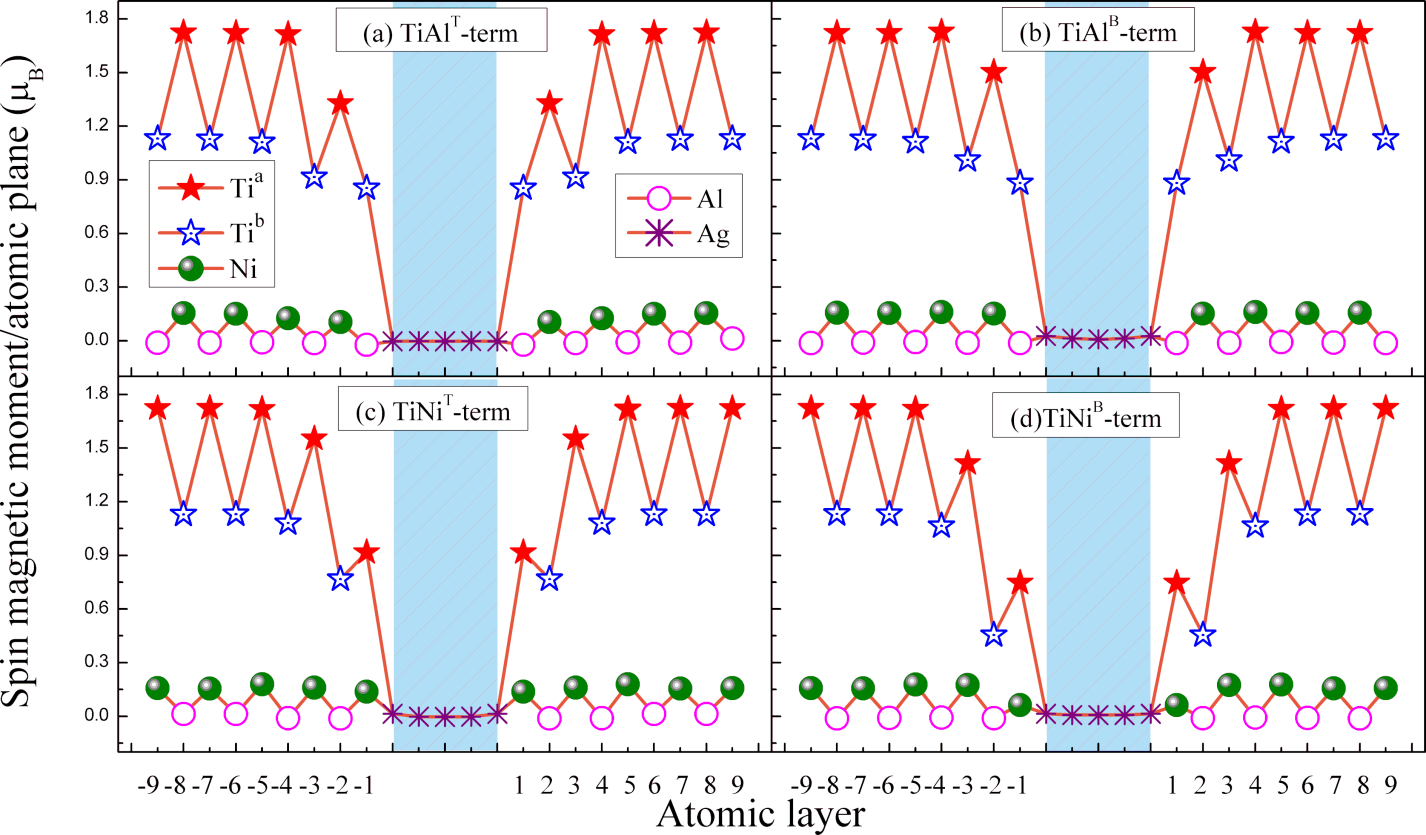
The calculated atomic spin magnetic moment of Ti_2_NiAl/Ag/Ti_2_NiAl CPP-SV at different atomic layers. (a) TiAl^T^-terminated structure, (b) TiAl^B^-terminated structure, (c) TiNi^T^-terminated structure and (d) TiNi^B^-terminated structure.

In order to study the magnetic behavior of Ti_2_NiAl/Ag/Ti_2_NiAl CPP-SV, we calculated the spin-resolved atom magnetic moment of each layer of the device with various atomic-terminated interfaces, which are shown in Figure 2. It can be seen that the magnetic moment of interfacial Ti^b^ atoms in the TiAl^T^-terminated (see Figure 2a) and the TiAl^B^-terminated structure (see Figure 2b) suffer from reduction compared to its value in bulk Ti_2_NiAl. Besides, magnetic moments of interfacial Ti^a^ and interfacial Ni atoms in TiNi^T^ terminated structures (see Figure 2c) also decrease, and they become lower in TiNi^B^-terminated structures (see Figure 2d). This reveals that, for an interface containing Ti and Ni atoms, the hybridization between interfacial Ti and Ni atoms in the TiNi^B^-terminated structure is stronger than those in the TiNi^T^-terminated structure. The total interfacial magnetic moments of TiAl^T^ and TiAl^B^ terminated structures are 0.831μ_B_ and 0.871μ_B_, respectively. The TiNi^T^-terminated structure has the highest total interfacial magnetic moment of 1.06μ_B_, while the TiNi^B^-terminated structure owns the lowest total interfacial magnetic moment of 0.81μ_B_. In addition, when Ti^a^, Ti^b^ and Ni are in the deep layer of the heterostructure, their magnetic moments are close to the values in Ti_2_NiAl bulk, indicating that interfacial effects have a minor influence on the magnetic moment of deep-layer atoms. The magnetic property of Al atoms can be explained by the Ruderman–Kittel–Kasuya–Yosida (RKKY) indirect exchange mechanism. According to the RKKY mechanism, the magnetic coupling among d-electron atoms is transferred through the conduction electrons, and the cooperative magnetic states would exhibit ferromagnetic or antiferromagnetic alignment of the moments largely dependent upon the interatomic distances. Because the Al atom is a typical conduction sp-electron atom, it can continually exchange with local d-electrons of the nearest transition metal and serves as a bridge in hybridization between local d-electrons atoms. Hence, the atomic magnetic moment of the Al atom always presents a small negative value.

**Figure 3:**
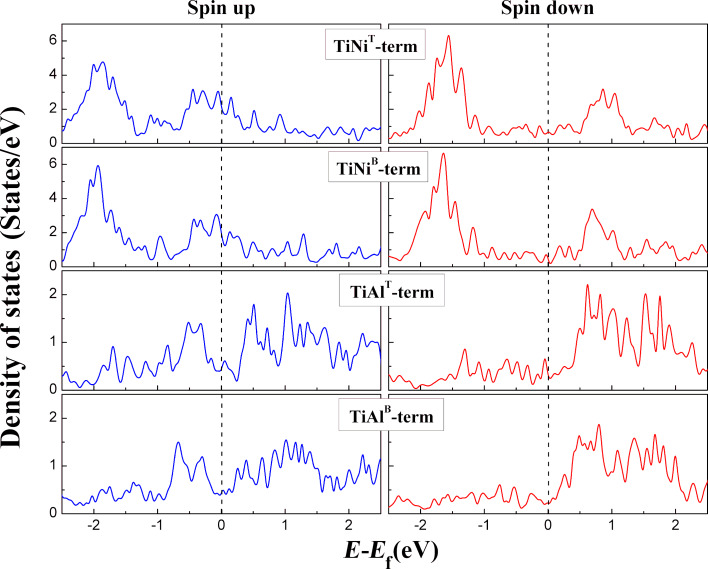
Density of states of the interface of the Ti_2_NiAl/Ag/Ti_2_NiAl device with different atomic terminated interface.

Because the interface spin polarization (ISP) plays an important role in determining the performance of a spin-dependent device, the interfacial electronic structure is calculated and exhibited in Figure 3, where the left panel and right panel indicate the interfacial electronic structure in spin up and spin down channels, respectively. It was observed that the spin down half-metallic energy gap in bulk Ti_2_NiAl is destroyed completely in various kinds of atomic-terminated interfaces of the Ti_2_NiAl/Ag/Ti_2_NiAl device, which could be attributed to the appearance of interface states [27–28]. As for interfaces containing Ti and Ni atoms, spin up and spin down density of states at the Fermi level in the TiNi^T^-terminated structure are close to those in the TiNi^B^-terminated structure, and the spin up density of states at the Fermi level are higher than spin down density of states at the Fermi level. As for interfaces containing Ti and Al atoms, spin up and spin down density of states at the Fermi level in both TiAl^T^ and TiAl^B^ terminated structures are much lower than in TiNi^T^ and TiNi^B^ terminated structures, and the spin up density of states at the Fermi level are comparable to spin down density of states at the Fermi level. The ISP can be defined as ISP = (*N*^↑^ − *N*^↓^)/(*N*^↑^ + *N*^↓^), where *N*^↑^ and *N*^↓^ represent the spin up and spin down contributions to the total density of states (DOS) at the Fermi level, respectively. Table 1 shows that the calculated ISP of the TiAl^B^-terminated structure is ≈30%, and it decreases to ≈20% in the TiAl^T^-terminated structure, which is the lowest ISP value. However, the TiNi^T^-terminated structure has a high ISP of ≈42%, and the TiNi^B^-terminated structure possesses the largest ISP of ≈55%.

**Table 1:** The calculated interface spin polarization (ISP), transmission coefficients at the Fermi level, and magnetoresistance ratio (MR) of the Ti_2_NiAl/Ag/Ti_2_NiAl device with various atomic terminations.

Termination	ISP	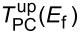	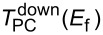	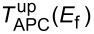	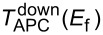	MR

TiNi^T^	≈42%	0.1067	0	0.1657 × 10^−6^	0.1869 × 10^−6^	3.03 × 10^5^
TiNi^B^	≈55%	0.1152	0	0.2041 × 10^−6^	0.1472 × 10^−6^	3.28 × 10^5^
TiAl^T^	≈20%	0.0408	0	0.2243 × 10^−6^	0.1991 × 10^−6^	9.64 × 10^4^
TiAl^B^	≈30%	0.0991	0	0.7354 × 10^−6^	0.7486 × 10^−6^	6.67 × 10^4^

**Figure 4:**
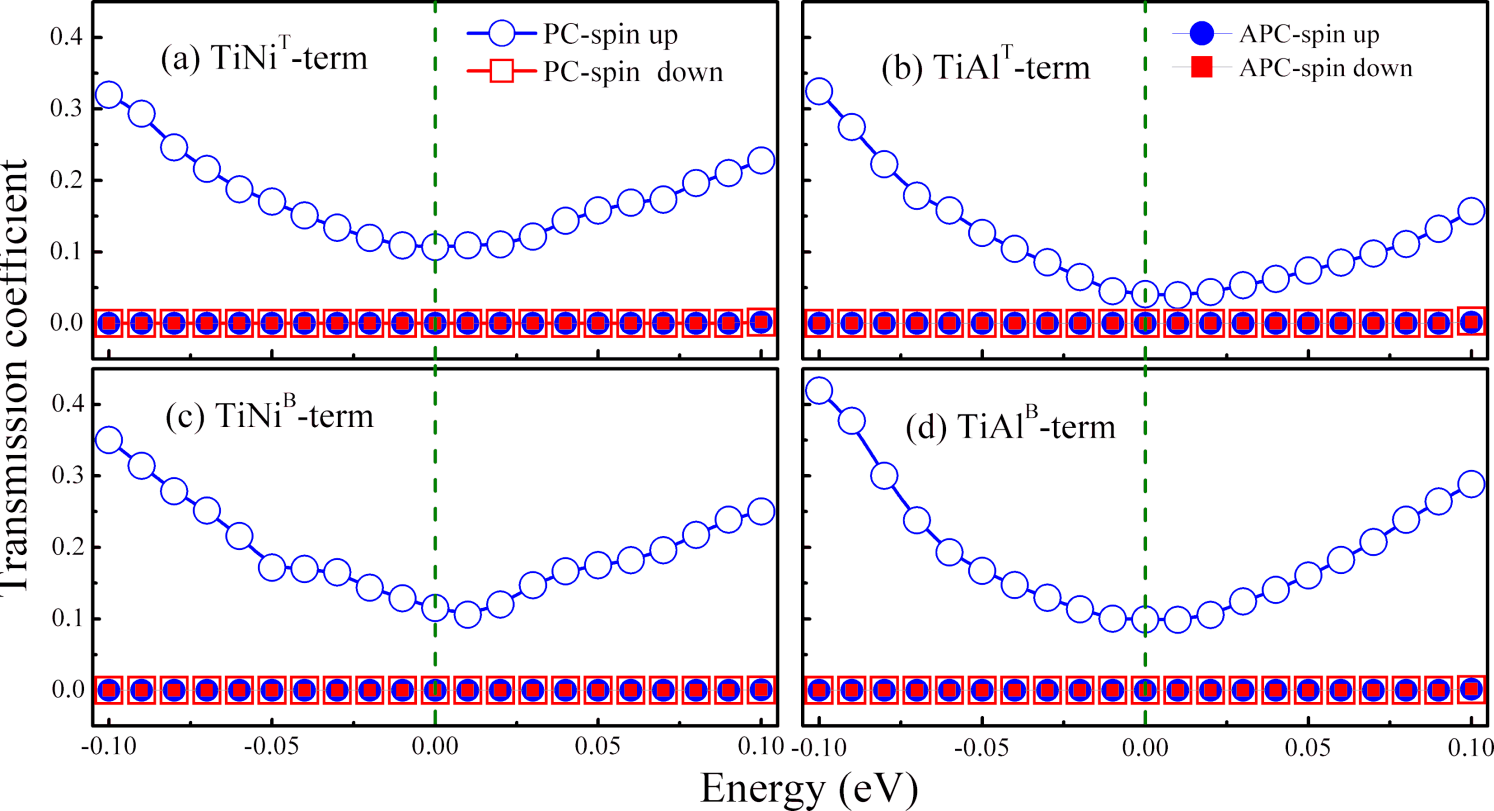
Transmission coefficient versus electron energy in the parallel magnetization configuration (PC) and antiparallel magnetization configuration (APC) of the Ti_2_NiAl/Ag/Ti_2_NiAl device with different atomic terminated interfaces. The dashed line is the Fermi level. (a) TiNi^T^-terminated structure, (b) TiAl^T^-terminated structure, (c) TiNi^B^-terminated structure and (d) TiAl^B^-terminated structure.

As for our two-probe device, the spin-dependent transmission coefficient *T*^σ^(*E*) can be calculated employing *T*^σ^(*E*) = *Tr*[Г_L_*G*^R^Г_R_*G*^A^], where Г_L_ and Г_R_ are the coupling matrix of the left and right electrode, respectively; *G*^R^ and *G*^A^ are the retarded and advanced Green’s function of the central region, respectively; and σ is the spin direction, spin up or spin down. A two magnetization configuration is considered, thus the two electrodes of the device are in parallel magnetization configuration (PC) and in antiparallel magnetization configuration (APC). The spin-dependent electron transmission curves of spin up and spin down channels of the device with different atomic terminated interfaces in PC and APC are calculated and shown in Figure 4, where the green dashed line indicates the Fermi level, which is set to zero. It can be seen that in APC, for all four kinds of interface structures, both the spin up and spin down channels, possess a very small transport coefficient at the Fermi level. This indicates that the spin up and spin down channels are blocked when the device is in the APC state, i.e., the spin-polarized current cannot be detected, and the device is turned off. When the device is in the PC state, a spin up transmission coefficient at the Fermi level (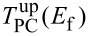) is much higher than the spin down transmission coefficient at the Fermi level (
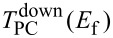
). This reveals that when the device is in the PC state, the spin up channel is unlocked and spin up electrons, which are regarded as the majority of spin electrons, can flow from the left electrode to the right electrode through a Ag spacer. In contrast, the spin down channel is still closed, and the spin down electrons, which are regarded as the minority of spin electrons, are suppressed. Hence, spin-polarized current can be detected when the device is in the PC state, and it is mainly dominated by spin up electrons, and the device is in a “turn on” mode. As for the device with an interface containing Ti and Ni atoms, in the PC state, the value of 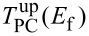 in the TiNi^T^-terminated structure (0.1067) is comparable to the value in the TiNi^B^-terminated structure (0.1152). In the APC state, the values of 
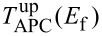
 and 
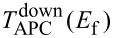
 in the TiNi^T^-terminated structure are also close to the values in the TiNi^B^-terminated structure. Besides, as for the device with an interface containing Ti and Al atoms, the value of 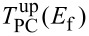 in the TiAl^T^-terminated structure is 0.0408, and it increases to 0.099 in the TiAl^B^-terminated structure. Nevertheless, it can be found that the device with the TiNi-terminated interface possesses a higher spin up transmission coefficient than the TiAl-terminated interface.

**Figure 5:**
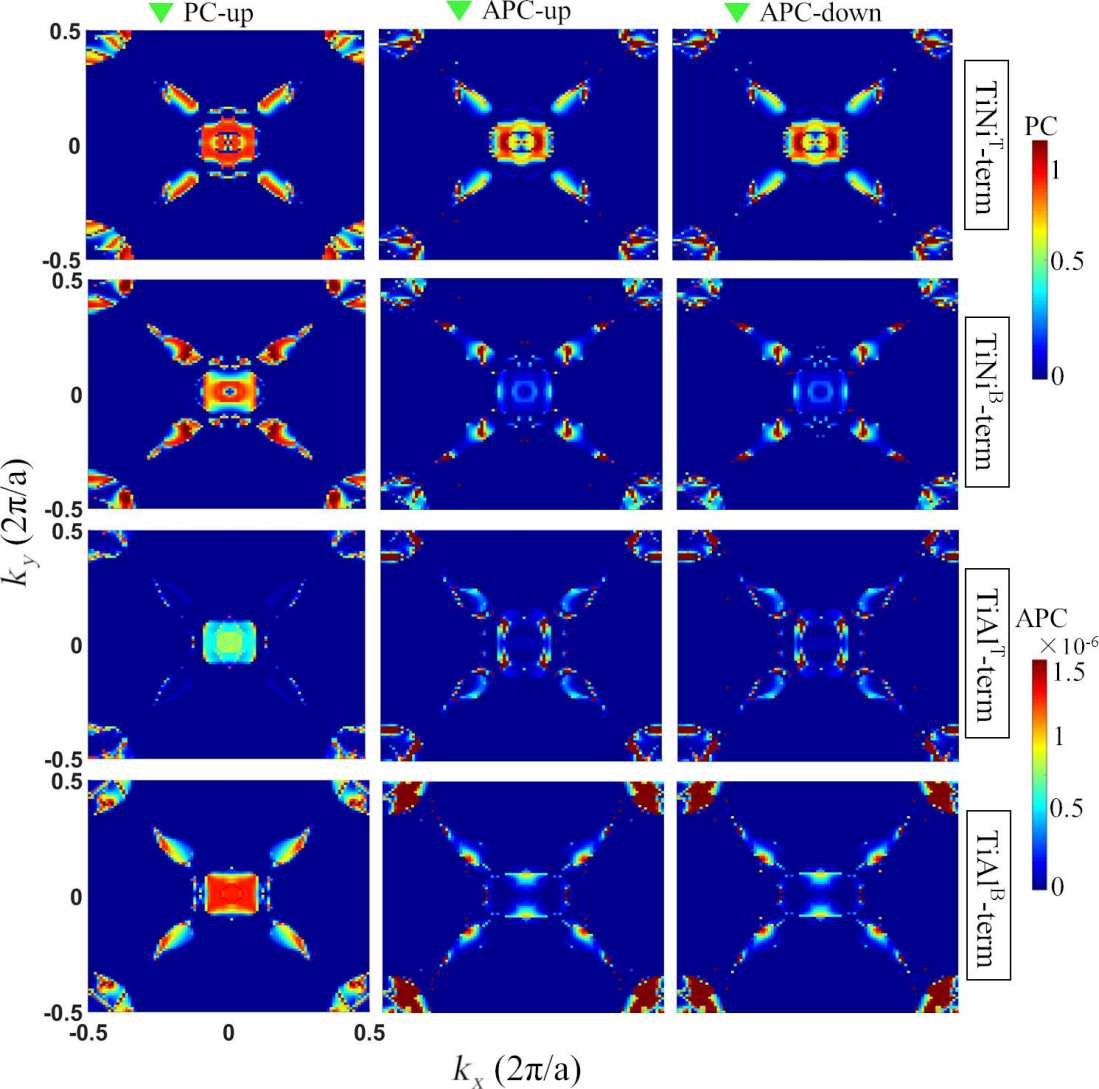
The *k*_//_-resolved transmission coefficients at the Fermi level of the Ti_2_NiAl/Ag/Ti_2_NiAl CPP-SV with different atomic terminated interfaces.

In order to further exhibit the spin-transport behavior of the device, the transmission coefficient at the Fermi level of various kinds of structures in the two-dimensional Brillouin zone, which is perpendicular to the spin-transport direction, was calculated. In Figure 5, the contour plots of the transmission coefficient at the Fermi level are shown as a function of *k_x_* and *k_y_* and indicate the transport behavior of spin up electrons of the device in the PC state. The plots in the middle column and the right column indicate the transport behavior of spin up and spin down electrons, respectively, of the device in the APC state. There are two color bars in Figure 5, and the upper color bar and the lower color bar represent the amplitude of the transport ability at various (*k_x_*, *k_y_*) points when the device is in the PC and APC state, respectively. It was observed that when the device has different atomic terminated interfaces in the PC state, the magnitude of the hot spots in the spin up channel is much stronger than those in the spin up and spin down channels of the device in APC. This reveals that when the device is in the APC state, the transport ability of spin up and spin down electrons is inhibited, and spin-polarized electrons are less likely to travel from the left electrode to the right electrode. Besides, in the PC state the magnitude of the spin up channel of the TiNi^T^-terminated structure is comparable to that of the TiNi^B^ and TiAl^B^ structures, while it becomes weaker in the TiAl^T^ termination. This reveals that when the device in the PC state, the spin up electrons of the TiNi^T^, TiNi^B^ and TiAl^B^ terminated structures have a similar intensity of transport ability, while the transport ability of the spin up electrons of the TiAl^T^ terminated structure suffers from deterioration. Now, the situation when the device is in the APC state will be discussed. It can be seen that in the APC state, the transmission spectra of the spin up channel are nearly the same as that of the spin down channel for all kinds of atomic terminated structures, revealing that the transport ability of the spin up electrons is close to that of the spin down electrons. This results in the consequence that when the device is in the APC state, it is difficult to identify the spin up current and the spin down current from the total spin-polarized current.

As one the most significant parameters in spintronics devices, the magnetoresistance (MR) ratio (when the device at equilibrium) can be calculated by

[1] $$MR=\left|\frac{T_{\text{PC}}(E_{\text{f}})-T_{\text{APC}}(E_{\text{f}})}{\min\left(T_{\text{PC}}(E_{\text{f}}),T_{\text{APC}}(E_{\text{f}})\right)}\right|\;,$$

where *T*_PC_(*E*_f_) and *T*_APC_(*E*_f_) indicate the total transmission coefficient at the Fermi level in the PC and APC states, where


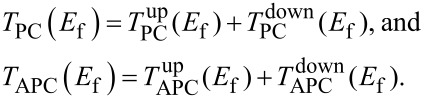


The MR ratios of the Ti_2_NiAl/Ag/Ti_2_NiAl device with various kinds of atomic terminated interfaces were calculated and listed in Table 1. It can be seen that the MR ratios of all the structures exceed ≈10^4^ order of magnitude. The device with a TiAl^B^ interface shows a high MR ratio of 6.67 × 10^4^, and the MR ratio is boosted to a higher value of 9.64 × 10^4^ for the TiAl^T^ interface structure. This reveals that for the Ti_2_NiAl/Ag/Ti_2_NiAl device with an interface containing Ti and Al atoms, the Heusler layer sits on the top site of the Ag atom and can produce larger a MR ratio than when it sits at the bridge site of the Ag atom. On the other hand, the MR ratio of the device with the TiNi^T^ terminated interface reaches up to 3.03 × 10^5^, and the MR ratio is further enhanced to an ultrahigh value of 3.28 × 10^5^ in the device with the TiNi^B^ terminated interface. Therefore, it can be deduced that the Ti_2_NiAl/Ag/Ti_2_NiAl device with the interface containing Ti and Ni atoms generally results in a higher MR ratio than that with interfaces containing Ti and Al atoms. Additionally, the device with a TiNi^B^-terminated interface possesses the largest MR value and can be regarded as a promising candidate for furture spintronics devices.

## Conclusion

By employing first principles calculations combined with the nonequilibrium Green’s function, we studied the interfacial magnetic properties, interfacial electronic structure and spin transport properties of a Ti_2_NiAl/Al/Ti_2_NiAl CPP-SV and four structures with different atomic terminated interfaces were modeled. Our calculation revealed that the magnetic moments of atoms located at the interface suffer a decrease, while the magnetic moments of atoms that sit at deep layers are close to corresponding values in bulk Ti_2_NiAl. The TiNi^T^-terminated structure possesses a high ISP of ≈42%, and the TiNi^B^-terminated structure has the largest ISP of ≈55%. The total transmission coefficient at the Fermi level mainly comes from the contribution of the spin up electrons, which is regarded as the majority of spin electrons. In the PC state, the spin up transmission coefficients of TiNi^T^ and TiNi^B^ terminated structures are higher than that of TiAl^T^ and TiAl^B^ terminated structures. The MR ratios of the device with four different interfaces, i.e., TiAl^T^, TiAl^B^, TiNi^T^ and TiNi^B^ terminated interfaces, have been calculated. Our calculation reveals that the device with TiNi^B^-terminated structure possesses the largest MR ratio of 3.28 × 10^5^, a value that is much higher than other CPP-SV devices such as Fe_4_N/Ag/Fe_4_N and Co_2_MnAl/Ag/Co_2_MnAl. Such a high MR ratio could be attributed to the complete spin-polarized Ti_2_NiAl bulk and high spin polarization at the interface of the device. Therefore, Ti_2_NiAl/Ag/Ti_2_NiAl CPP-SV has great application potential in spintronic devices.

## Simulation Details

The Ti_2_NiAl/Ag/Ti_2_NiAl device with four different atomic terminated interfaces was geometrically optimized by utilizing a density functional theory (DFT)-based Vienna ab-initio simulation package (VASP) [31–32]. Ti (3*d*^2^4*s*^2^), Ni (3*d*^8^4*s*^2^), Al (3*s*^2^3*p*^1^) and Ag (4*d*^10^5*s*^1^) were chosen to be the valence electron configurations. The exchange-correlation interaction is described by the Perdew–Burke–Ernzerhof (PBE) generalized gradient approximation (GGA). A Monkhorst–Pack grid of 13 × 13 × 1 for *k*-point sampling, a self-consistent field (SCF) convergence criterion of 1 × 10^−5^ eV, and a plane-wave basis cutoff energy of 550 eV were applied. The Keldysh nonequilibrium Green’s function (NEGF) theory, as implemented in Nanodcal package [33–34], was employed to investigate the spin-transport properties of the Ti_2_NiAl/Ag/Ti_2_NiAl device. In our calculations of transport properties, the number of Monkhorst–Pack k-space grids of the left and right electrode is 10 × 10 × 100, and that of the central scattering region is 10 × 10 × 1, where the self-consistent calculations are limited to 10^−5^ Hartree tolerance.
